# Highly Aggressive Relapsing Course of Myelin Oligodendrocyte Glycoprotein (MOG) Antibody-Associated Disease Requiring Intensive Care Unit Treatment and Quadruple Immunotherapy

**DOI:** 10.7759/cureus.105417

**Published:** 2026-03-17

**Authors:** Deepak Sharma, Wiebke Böhmer, Christian P Kamm, Lara Diem

**Affiliations:** 1 Neurology, Luzerner Kantonsspital, Luzern, CHE; 2 Neurology, Klinik Lengg, Zürich, CHE; 3 Neurology, Inselspital Bern, Bern, CHE

**Keywords:** delayed seroconversion, ivig, live-cell binding assay, mogad, mog antibody-associated disease, quadruple immunotherapy, rituximab, tocilizumab, treatment-refractory demyelination

## Abstract

Myelin oligodendrocyte glycoprotein antibody-associated disease (MOGAD) is an autoimmune-mediated demyelinating disorder of the central nervous system (CNS). Clinical manifestations and disease courses range from involvement of the optic nerve, spinal cord, or brainstem to acute disseminated encephalomyelitis (ADEM), and from monophasic to relapsing or progressive forms. Diagnosis can be challenging; repeated testing is sometimes required to establish the diagnosis, particularly in atypical clinical presentations. First-line therapy in the acute setting consists of corticosteroids, whereas steroid-sparing agents are used for long-term management in relapsing cases. Various therapeutic strategies, particularly in aggressive courses, have been described in case reports. We report a challenging case of a 34-year-old woman with a severe relapsing disease course requiring intensive care unit (ICU) support. Anti-myelin oligodendrocyte glycoprotein-immunoglobulin G (anti-MOG IgG) was detected only after repeated testing using different assays. Brain biopsy identified an unidentified viral pathogen of uncertain clinical relevance. The disease demonstrated marked refractoriness to standard therapies.

## Introduction

Myelin oligodendrocyte glycoprotein antibody-associated disease (MOGAD) is an inflammatory demyelinating disorder of the central nervous system (CNS) that is clinically and radiologically distinct from multiple sclerosis (MS) and neuromyelitis optica spectrum disorder (NMOSD) [1]. The diagnosis relies on the detection of serum anti-MOG immunoglobulin G (IgG) using cell-based assays (CBAs). However, relevant variability between different assay formats, particularly between fixed and live CBAs, has been reported and may result in discrepant serological findings [2-5].

Management of MOGAD can be challenging in patients with severe or treatment-refractory disease courses. Acute attacks are typically treated with high-dose corticosteroids (CS), while escalation therapies, such as plasma exchange (PLEX) or intravenous immunoglobulins (IVIG), are frequently required in steroid-refractory cases. In patients with relapsing or aggressive disease, long-term immunotherapy is often considered, although evidence guiding the optimal maintenance strategy remains limited, and many treatments are currently used off-label [6-11].

Here, we report a case of a fulminant demyelinating disease course initially diagnosed as seronegative NMOSD because repeated testing with a fixed CBA for MOG antibodies remained negative. The diagnosis of MOGAD was ultimately established by a live CBA after extensive diagnostic evaluation, including a brain biopsy.

## Case presentation

Clinical findings 

A 34-year-old female presented to the emergency department with no known medical conditions, medications, or allergies, and no relevant family history.

The first relapse began with paresthesia of the left hand and foot. Approximately one week later, progressive blurred vision in the left eye developed, and two weeks after symptom onset, the patient presented with near-complete bilateral visual loss. Neurological examination revealed a left-sided relative afferent pupillary defect (RAPD), sensory deficits below thoracic level 10 (Th10), and an unsteady gait. The patient was fully alert and oriented. Following inpatient relapse treatment with high-dose CS and PLEX, visual acuity gradually improved. The patient was subsequently discharged to the outpatient setting for initiation of therapy with a suspected diagnosis of aquaporin-4 immunoglobulin G-negative NMOSD (AQP4-IgG-negative NMOSD).

The second relapse occurred three weeks after hospital discharge and was characterized by profound fatigue, cognitive impairment, recurrent visual deterioration, abdominal hypesthesia, and vertigo. Following emergency hospitalization, the patient’s neurological status rapidly deteriorated within several days, with a decline in consciousness to a Glasgow Coma Scale (GCS) score of 6, necessitating immediate transfer to the ICU. As protective airway reflexes remained preserved, endotracheal intubation was not performed.

Severe cognitive deficits were observed, including aphasia with non-fluent speech accompanied by semantic and phonemic paraphasias, as well as dysarthrophonia. The patient was arousable to moderate painful stimuli and moved all extremities symmetrically.

Following various relapse treatments, gradual clinical stabilization was achieved. The patient was subsequently transferred to a three-month inpatient neurorehabilitation program involving multidisciplinary management by neurology, ophthalmology, and psychiatry. At discharge from rehabilitation, bilateral visual function had fully recovered. However, distal pallhypesthesia and persistent paresthesias remained, accompanied by significant physical and cognitive fatigue that resulted in the inability to return to work. Prior to the illness, the patient had been employed as a project manager.

**Figure 1:**
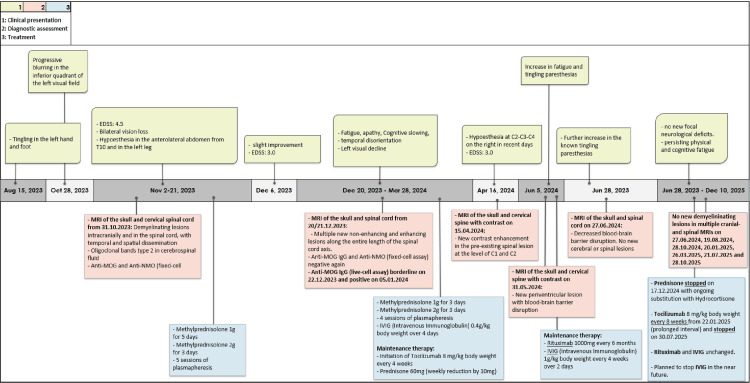
Case report timeline. Summary of clinical presentation, diagnostic assessment, and treatment. AQP4-IgG: aquaporin 4-immunoglobulin G, EDSS: Expanded Disability Status Scale, FLAIR: fluid-attenuated inversion recovery, MOG: myelin oligodendrocyte glycoprotein, MRI: magnetic resonance imaging, NMO: neuromyelitis optica Image made using Microsoft Excel (Microsoft® Corp., Redmond, WA).

**Figure 2:**
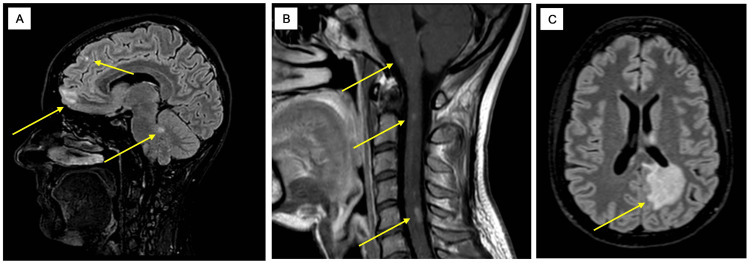
MRI cranium and whole spine, native and with contrast agent. (A) 31.10.2023 T2 FLAIR axial: multiple demyelinating lesions located in the cortex, left frontobasal region, left frontal region, and cerebellum (yellow arrows). (B) 31.10.2023 T1 with contrast agent sagittal: multiple contrast-enhancing demyelinating lesions located in the spinal cord (yellow arrows). (C) 20.12.2023 T2 FLAIR axial: new FLAIR hyperintense confluent demyelinating lesion predominantly located periventricularly in the left parietal region (yellow arrow). FLAIR: fluid-attenuated inversion recovery, MRI: magnetic resonance imaging

A more detailed depiction of the clinical course, relapse dynamics, and diagnostic assessments is provided in the graphical representations (Figures 1-2).

Diagnostic assessment 

During the first relapse, magnetic resonance imaging (MRI) of the brain and spinal cord demonstrated more than 10 demyelinating lesions both intracranially and within the spinal cord, showing temporal and spatial dissemination. Cerebrospinal fluid (CSF) analysis revealed mild lymphocytic pleocytosis (cell count: 14), slightly elevated protein levels, and type II oligoclonal bands. Initial testing for anti-MOG IgG using a fixed CBA (FCBA), as well as testing for AQP4-IgG, was negative, leading to a provisional diagnosis of seronegative NMOSD. An atypical manifestation of MS was considered as a differential diagnosis.

During the second relapse, repeat testing for anti-MOG IgG using the FCBA again yielded a negative result. Cranial MRI demonstrated marked progression with multiple hyperintense T2-weighted/fluid-attenuated inversion recovery (FLAIR) lesions, predominantly periventricular and parietal on the left and bilaterally in the temporopolar regions. Additional lesions were observed in the corpus callosum, dorsal thalami, pons, brainstem, and cerebellum. Contrast enhancement was particularly evident in the left periventricular and parietal lesions and in the temporopolar regions. Spinal MRI revealed multiple non-enhancing and enhancing lesions that had developed since the previous examination along the entire length of the spinal cord axis. CSF analysis showed lymphocytic pleocytosis (cell count: 43) and borderline type II oligoclonal bands.

Because of ongoing disease activity despite relapse treatment with CS and PLEX, a brain biopsy was performed, confirming an active demyelinating process. Histopathological examination of the brain tissue revealed numerous histiocytes and reactive astrocytes, consistent with an acute demyelinating lesion. At an early stage of the diagnostic work-up, the presence of JC virus (JCV)/polyomavirus 2-related material in the biopsy specimen was suspected. However, subsequent analyses did not confirm this finding, and JCV DNA testing was negative. Consequently, the presence and potential relevance of the virus remain unclear. Importantly, there was no histological evidence of progressive multifocal leukoencephalopathy (PML) [12]. 

Finally, testing with a live CBA (LCBA) performed at a specialized laboratory yielded a positive result, thereby confirming the diagnosis of MOGAD.

Therapeutic intervention 

During the first relapse, intravenous methylprednisolone at 1 g/day for five days was initiated but produced no improvement. Therapy was subsequently escalated to 2 g/day, administered intravenously over eight days, without symptomatic relief. Five sessions of PLEX were then performed, resulting in significant clinical improvement.

During the second relapse, symptoms progressively worsened despite high-dose intravenous CS and consecutive PLEX. A five-day course of IVIG combined with an additional course of high-dose CS ultimately led to symptomatic improvement. Maintenance therapy with tocilizumab was initiated, and CS were gradually tapered. Rituximab (RTX) was considered but initially withheld due to the unclear viral findings in the brain biopsy.

Disease activity recurred despite ongoing immunomodulatory treatment with IVIG, CS, and tocilizumab (after the second administration). In response, immunotherapy was escalated to a quadruple regimen consisting of RTX 1000 mg every six months, continued tocilizumab at 8 mg/kg body weight every four weeks, IVIG, and CS. Thereafter, sustained clinical and radiological stability was achieved and maintained for 19 months. Subsequently, CS were tapered, the tocilizumab dosing interval was extended to every eight weeks, and tocilizumab was eventually discontinued without evidence of clinical or radiological relapse. Neuropathic pain was effectively managed with pregabalin and duloxetine.

The current therapeutic goal is to maintain disease stability with RTX monotherapy while tapering IVIG.

Follow-up and outcomes 

The patient remains clinically stable, with complete recovery of vision and normal findings on formal neurocognitive testing. However, severe fatigue persists, resulting in complete inability to work. Other neurological deficits, including sensorimotor impairments and gait instability, have resolved.

## Discussion

This case highlights three clinically relevant insights for the diagnosis and management of MOGAD. First, it illustrates the diagnostic challenges and limitations of current anti-MOG antibody assays, emphasizing the importance of repeated testing and awareness of methodological variability. Second, it demonstrates that MOGAD can follow an unexpectedly aggressive, relapsing course that may necessitate intensive care unit (ICU) support. Third, it underscores the therapeutic complexities of refractory disease, in which conventional immunotherapies may be insufficient and biologic or experimental strategies may be required.

These observations are further contextualized by published data on diagnostic reliability, disease course, and therapeutic approaches.

Diagnostic complexity and reliability of anti-MOG antibody assays

Comparative multicenter studies of anti-MOG IgG detection methods reveal significant performance differences between LCBAs and FCBAs, with LCBAs typically offering 5-15% higher sensitivity for low-titer or conformationally sensitive antibodies while preserving specificity >95%. Discordance rates between platforms reach 10-25% in clinically suspected cases, often due to variations in cell line transfection, antigen density, fixation artifacts disrupting epitopes, and laboratory-specific cutoff thresholds [2-5,13-15]. Serial testing thus detects seroconversion in up to 20% of initially negative patients, while switching from FCBA to LCBA confirms MOGAD in many with compatible MRI and clinical features, as occurred here after repeated fixed-cell negatives [3-5,14,15]. Updated diagnostic criteria therefore recommend assay diversification, repeat sampling during active disease, and expert consultation to minimize false negatives and avoid misdiagnosis as seronegative NMOSD [14-16]. These findings mirror the delayed seroconversion in this patient, in whom repeated fixed-cell testing remained negative until a live-cell assay at a reference laboratory confirmed MOGAD.

Severity and relapsing nature of MOGAD

Prospective and retrospective cohorts have shifted the view of MOGAD from predominantly monophasic (relapse risk ~20-30% at two years) to frequently relapsing, with 40-60% experiencing recurrence over five years and annualized relapse rates of 0.2-0.5, most commonly recurrent optic neuritis (60-70% of events). Relapses accrue greater Expanded Disability Status Scale (EDSS) progression (mean +1.5 points at five years vs. monophasic) and face higher risks of permanent visual or motor deficits [6,7,17,18]. Severe attacks affect 5-15%, including brainstem syndromes with respiratory compromise requiring ventilation in 2-5% and ICU admission, mirroring this patient's coma and GCS drop to 6 [7,8]. Despite this, recovery potential remains high (80-90% achieve EDSS ≤3 long-term) with timely control, underscoring the need for vigilant monitoring and low-threshold escalation [6,7,17,18]. The five relapses within ten months and the need for intensive care in this case are in line with these high-risk phenotypes described in the literature, while the eventual full visual and cognitive recovery illustrates the potential for a good outcome when disease activity is ultimately controlled, and supports prolonged treatment of relapses, including ICU care.

Treatment resistance and therapeutic considerations

Across observational cohorts, high-dose CS and PLEX are effective for most acute attacks, but incomplete or absent responses are reported in a substantial minority of severe episodes, motivating early escalation to rescue treatments [6-9,19,20]. For relapse prevention, several comparative studies suggest that IVIG is associated with the lowest annualized relapse rates and highest relapse-free proportions, outperforming mycophenolate mofetil and showing more consistent benefit than many anti-CD20 regimens in MOGAD, particularly when introduced earlier in the disease course [6,19,21]. In refractory patients, interleukin-6 (IL-6) receptor inhibitors such as tocilizumab and B-cell-depleting monoclonal antibodies have shown additional reductions in relapse risk, although responses are heterogeneous and often partial, highlighting disease-specific differences compared with AQP-4-positive NMOSD [8,9,19]

More recently, small case series and reports have explored autologous hematopoietic stem cell transplantation and chimeric antigen receptor T-cell-based strategies in highly aggressive, treatment-resistant antibody-mediated demyelination, including MOGAD, with promising but still preliminary efficacy signals and unresolved questions about long-term safety and optimal patient selection [8,20]. The need in this patient for quadruple immunotherapy with RTX, tocilizumab, IVIG, and CS, followed by careful de-escalation once stability is achieved, is consistent with this broader literature trend toward escalation-oriented, individualized regimens in severe, relapsing MOGAD.

## Conclusions

In summary, this case highlights key challenges in the diagnosis and management of MOGAD. It underscores the diagnostic complexity of current anti-MOG antibody assays and the importance of repeated testing across complementary methodologies when clinical suspicion persists despite initial seronegativity. The case also illustrates that MOGAD can follow an unexpectedly aggressive, relapsing course, occasionally requiring intensive care support. Furthermore, it demonstrates the therapeutic difficulties of treatment-refractory disease, where conventional immunotherapies may be insufficient and timely escalation to targeted biologic or experimental strategies becomes essential. Overall, this case emphasizes the value of assay diversification, early biologic escalation in steroid-unresponsive disease, and coordinated multicenter efforts to refine diagnostic precision and guide therapeutic decision-making in this evolving disorder.
